# Analysis of 44 Vibrio anguillarum genomes reveals high genetic diversity

**DOI:** 10.7717/peerj.10451

**Published:** 2020-12-03

**Authors:** Mie Johanne Hansen, Egle Kudirkiene, Inger Dalsgaard

**Affiliations:** 1 National Institute of Aquatic Resources Technical University of Denmark, Kongens Lyngby, Denmark; 2 Department of Veterinary and Animal Sciences, University of Copenhagen, Frederiksberg C, Denmark

**Keywords:** Vibrio anguillarum, Genomes, Vibriosis, Virulence factors, Rainbow trout, Plasmids, Acquired antibiotic resistance genes

## Abstract

Vibriosis, a hemorrhagic septicemic disease caused by the bacterium *Vibrio anguillarum*, is an important bacterial infection in Danish sea-reared rainbow trout. Despite of vaccination, outbreaks still occur, likely because the vaccine is based on *V. anguillarum* strains from abroad/other hosts than rainbow trout. Information about the genetic diversity of *V. anguillarum* specifically in Danish rainbow trout, is required to investigate this claim. Consequently, the aim of the present investigation was to sequence and to characterize a collection of 44 *V. anguillarum* strains obtained primarily from vibriosis outbreaks in Danish rainbow trout. The strains were sequenced, de novo assembled, and the genomes examined for the presence of plasmids, virulence, and acquired antibiotic resistance genes. To investigate the phylogeny, single nucleotide polymorphisms were identified, and the pan-genome was calculated. All strains carried *tet(34)* encoding tetracycline resistance, and 36 strains also contained *qnrVC6* for increased fluoroquinolone/quinolone resistance. But interestingly, all strains were phenotypic sensitive to both oxytetracycline and oxolinic acid. Almost all serotype O1 strains contained a pJM1-like plasmid and nine serotype O2A strains carried the plasmid p15. The distribution of virulence genes was rather similar across the strains, although evident variance among serotypes was observed. Most significant, almost all serotype O2 and O3 strains, as well as the serotype O1 strain without a pJM1-like plasmid, carried genes encoding piscibactin biosynthesis. Hence supporting the hypothesis, that piscibactin plays a crucial role in virulence for pathogenic strains lacking the anguibactin system. The phylogenetic analysis and pan-genome calculations revealed great diversity within *V. anguillarum*. Serotype O1 strains were in general very similar, whereas considerable variation was found among serotype O2A strains. The great diversity within the *V. anguillarum* serotype O2A genomes is most likely the reason why vaccines provide good protection from some strains, but not from others. Hopefully, the new genomic data and knowledge provided in this study might help develop an optimized vaccine against *V. anguillarum* in the future to reduce the use of antibiotics, minimize economic losses and improve the welfare of the fish.

## Introduction

*Vibrio (Listonella) anguillarum* is a pathogenic bacterium that causes vibriosis, a hemorrhagic septicemic disease, in many species of fish and shellfish (Hickey & Lee, 2017; Larsen, 1990). *V. anguillarum* can induce infection as quickly as two days following initial exposure and turn lethal three days post-infection (Mikkelsen et al., 2007; Gram et al., 1999). Vibriosis is probably the oldest recognized bacterial fish disease, first documented in eels in 1718 (Bonaveri, 1761), and since then identified in at least 48 different species of fish (Pedersen, 1999). So far, 23 different O-serotypes of *V. anguillarum* (Sørensen & Larsen, 1986) have been described in the literature; however only serotypes O1-O3 and, to a lesser extent, O4 and O5, have been identified as causative agents of vibriosis in fish (Pazos et al., 1993; Larsen, Pedersen & Dalsgaard, 1994).

Rainbow trout (*Oncorhynchus mykiss*) comprise 95% of fish production in Danish aquaculture and are reared at both freshwater and marine farms (Danish Fisheries Agency, 2018). In 2017, 35.736 tons of rainbow trout were produced in Denmark, and the production increases every year (Danish Fisheries Agency, 2018). *V. anguillarum* is one of the most important bacterial pathogens in Danish marine farms and has, in recent years, also been a problem for freshwater farms (Pedersen et al., 2008). In Danish rainbow trout farming, it is primarily the O1 and O2A serotypes that cause disease (Pedersen, 1999), so fish are vaccinated against these serotypes (Alphaject 3000; Pharmaq, Norway) prior to transfer from freshwater farms to marine net cages. However, vibriosis outbreaks still occur among vaccinated fish, requiring treatment with antibiotics and leading to economic losses for the farmers (Pedersen et al., 2008). Thus, the vaccine does not seem to provide sufficient protection against vibriosis in rainbow trout in Danish fish farms. The reason for this suboptimal protection could be that the inactivated vaccine is based on *V. anguillarum* strains from abroad/other hosts than rainbow trout (Marana et al., 2019). Further and more detailed information about the specific genetic diversity in Danish *V. anguillarum* strains from rainbow trout is required to confirm this reason.

In April 2020, a total of 64 *V. anguillarum* genomes were available in GenBank. However, only 43 represented unique wildtype genomes. Of these, only nine genomes isolated from rainbow trout were serotype O1; similarly, only one isolate represented serotype O2. Consequently, the aim of this research was to sequence and characterize a collection of 44 *Vibrio anguillarum* strains with known serotypes, obtained primarily from vibriosis outbreaks in Danish rainbow trout, to expand our knowledge about the genotypic diversity of the taxa.

## Materials and Methods

A total of 44 *Vibrio anguillarum* strains (Table S1), available at the National Institute of Aquatic Resources culture collection, were selected for whole genome sequencing. Strains were identified as previously described (Larsen, Pedersen & Dalsgaard, 1994; Larsen & Olsen, 1991; Austin et al., 1995; Thompson et al., 2009) and selected based on host, serotype, location, water type, and year of isolation. The majority, 39 strains, were isolated from vibriosis outbreaks in Danish rainbow trout farms between 1978 and 2017. Five strains were isolated from other fish species, two from the European flounder (*Platichthys flesus*), one from the European eel (*Anguilla anguilla*), one from cod (*Gadus morhua*) and one from a Northern pike (*Esox lucius*); the latter was also the only strain not isolated in Denmark, but in Finland. The serotypes of the strains were distributed as follows: 19 isolates of serotype O1, 23 of serotype O2A, one serotype O2B, and one serotype O3.

### Sample preparation

The strains were grown in Veal Infusion Broth (Difco, Sparks, MD, USA) at 20 °C for 48 h and then inoculated on blood agar plates (Colombia agar base, Oxoid, Roskilde, Denmark) with 5% calf blood for incubation at 20 °C for 48 h. Genomic DNA was extracted with the QIAGEN QIAamp DNA mini kit (QIAGEN, Valencia, CA, USA) in accordance with the manufacturer’s instructions and immediately stored at −20 °C until further use. DNA quality was determined using the NanoDrop ND-1000 (Thermo Scientific, Waltham, MA, USA) and DNA concentration using the Qubit 2.0 fluorometer and Quant-iT dsDNA BR kit (Invitrogen, Carlsbad, CA, USA).

### Whole genome sequencing and assembly

The genomes were sequenced at Admera Health (South Plainfield, NJ, USA) using the Illumina Hiseq platform with a paired-end read length of 150 bp. Library construction with KAPA Hyper Prep Kit (Kapa Biosystems, Wilmington, MA, USA), sequencing, and data pipelining were performed in accordance with the manufacturer´s protocols. The raw reads were de novo assembled using the CLC Genomics Workbench 11.0.1 (https://www.qiagenbioinformatics.com/) and contigs smaller than 200 bp were discarded. The genomes were deposited in GenBank with the accession numbers summarized in Table S1.

### Single nucleotide polymorphisms

Single nucleotide polymorphisms (SNPs) were identified with the pipeline CSI Phylogeny 1.4 (https://cge.cbs.dtu.dk/services/CSIPhylogeny/) (Kaas et al., 2014) using the raw adapter and quality trimmed reads and the complete genome sequence of the O1 serotype *V. anguillarum* strain 775, isolated from a Coho salmon (*Oncorhynchus kisutch*) in 1974 in the United States of America, GenBank accession number GCA_000217675.1, as a reference (Naka et al., 2011; Gould, 1977). The analysis was run with default settings, and all indels were excluded. iTOL v4 (Letunic & Bork, 2016) was used to visualize the phylogenetic tree, including the metadata.

### Acquired antibiotic resistance genes

Acquired antibiotic resistance genes (ARGs) were identified using the pipeline ResFinder 3.0 (https://cge.cbs.dtu.dk/services/ResFinder/) (Zankari et al., 2012), available from the Center of Genomic Epidemiology, and thresholds were set to 75% similarity (ID) and 60% alignment length (coverage).

### Plasmids

The presence of plasmids in the strains was investigated using MyDbFinder 1.1 (https://cge.cbs.dtu.dk/services/MyDbFinder/). Nine known *V. anguillarum* plasmid sequences (Table S3) from the NCBI database were used to search for homologs in the assembled *V. anguillarum* genomes. Threshold limits for ID and coverage were set to 75% and 60%, respectively. For plasmid pJM1, and the pJM1-like plasmids p65, pM3_unnamed and p67vang, the ID was increased to 100% due to the high similarity of these plasmids.

### Virulence genes

Virulence factors are defined as genetic attributes that increase the chance to cause damage in a host, meaning molecules produced by bacteria, which augment their effectiveness and enable them to achieve colonization, immunoevasion, immunosuppression, or the ability to obtain nutrition from the host (Diard & Hardt, 2017). VirulenceFinder 1.5 (https://cge.cbs.dtu.dk/services/VirulenceFinder/) (Joensen et al., 2014) was used to screen for putative virulence factors using databases for *Listeria*, *Staphylococcus aureus*, *Escherichia coli* and *Enterococcus* with thresholds set at 85% for ID and 60% for coverage. Moreover, 240 virulence-related genes of *V. anguillarum* strain 775 (Naka et al., 2011; Castillo et al., 2017) and 10 of *V. anguillarum* strain RV22 (Balado et al., 2018) (Table S4) were used to identify homologs in the *V. anguillarum* genomes using MyDbFinder 1.1 (https://cge.cbs.dtu.dk/services/MyDbFinder/), with thresholds set at 80% for ID and 60% for the coverage.

### Pan-genome analysis

The pan-genome was calculated with Roary version 3.12.0 (Page et al., 2015), a high-speed stand-alone pan-genome pipeline, using GFF3 files produced by Prokka 1.12-beta (Seemann, 2014). The program was run using the default settings, which uses BLASTp for an all-against-all comparison with a percentage sequence identity of 95%. The hierarchical gene presence/absence tree created by Roary was visualized with Phandango (Hadfield et al., 2018) using the output.

### Antimicrobial sensitivity testing

All strains were subjected to antimicrobial susceptibility testing using the disc diffusion method in accordance with Clinical and Laboratory Standards Institute guidelines (CLSI, 2016). The strains were tested with oxytetracycline (30 µg) and oxolinic acid (2 µg) susceptibility discs (Oxoid, Basingstoke, the United Kingdom). Bacteria were cultured on Mueller-Hinton agar plates (SSI Diagnostic, Hillerød, Denmark) and incubated at 20 °C for 48 h, after which inhibition zones were measured.

## Results

### Genome properties

The 44 genomes were deposited in GenBank. The accession numbers and genome properties for the individual strains can be seen in Table S1. The number of contigs was high, between 881 and 2,198, but with excellent coverage from 252 to 355. The N50 varied from the relatively low 27.045 to the quite high 284.067. These numbers, as well as genome assembly evaluation with QUAST (Gurevich et al., 2013), demonstrated that the genomes were of varying but good quality. The size of the genomes ranged from 4.356.095 to 5.562.798 base pairs, the total number of genes from 4,436 to 6,304, the total number of CDS (coding sequences) from 4,364 to 6,225, the number of proteins from 4,225 to 6,025, and the number of RNAs from 72 to 92. The GC% was between 43,74 and 44,42.

### Phylogeny

**Figure 1:**
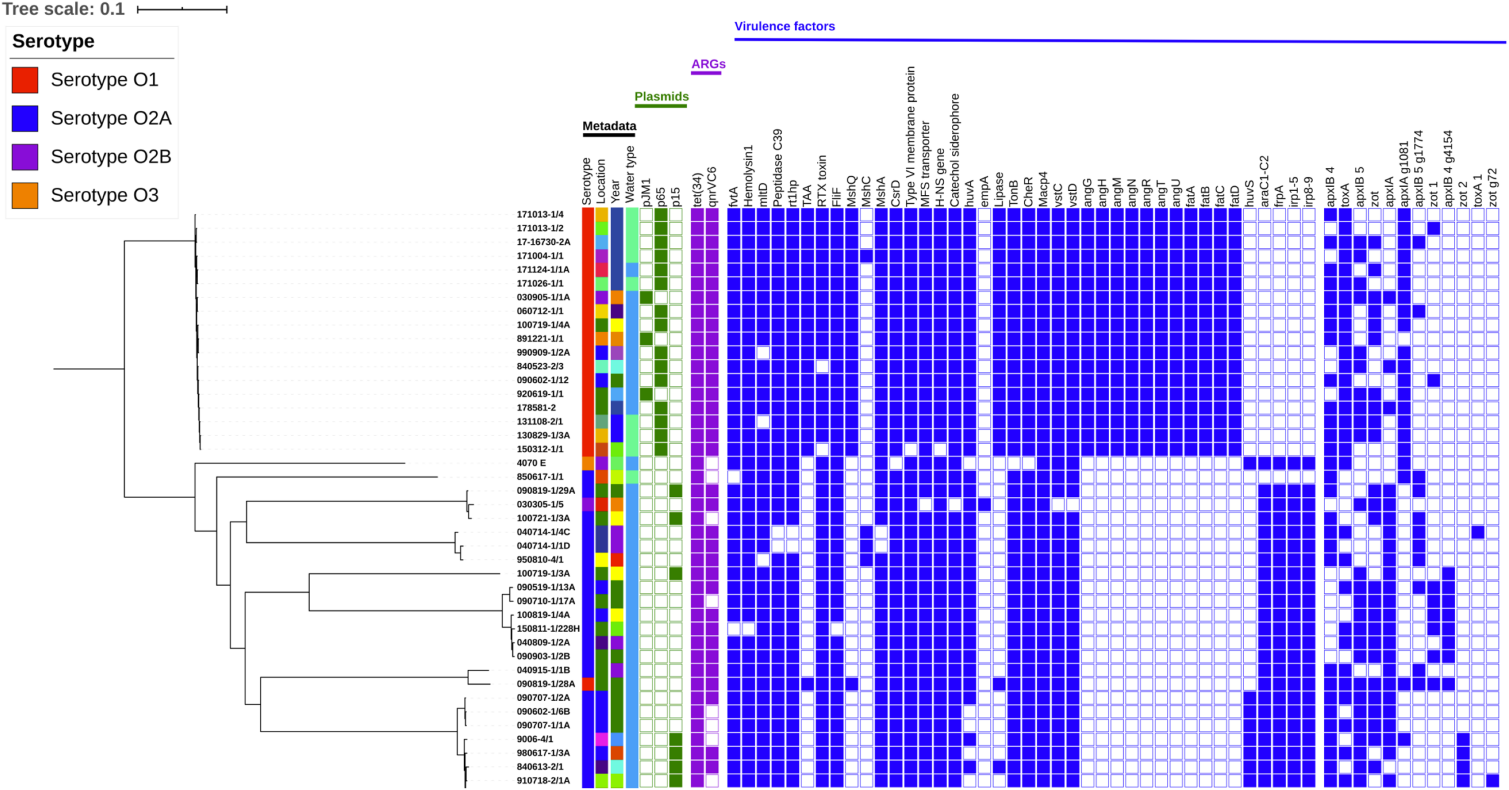
Phylogenetic tree including metadata. Phylogenetic tree including metadata constructed with iTOL v4 based on single nucleotide polymorphisms (SNPs) identified with the pipeline CSI Phylogeny 1.4, using the complete genome of *V. anguillarum* strain 775 as a reference. The first four columns with strain metadata represent serotype, location, year of isolation, and water type, respectively. The green cubes represent plasmid content; the purple cubes represent content of acquired antibiotic resistance genes and the blue cubes represent content of the putative virulence factors, which were not conserved amongst all the strains. Detailed metadata from each strain can be seen in Table S1, S2, S3 and S4. An enlarged copy of the phylogenetic tree, including bootstrap values, can be seen in Fig. S1.

The 44 genomes grouped into nine clusters in the SNP-based phylogenetic tree (Fig. 1). One cluster represented a monophyletic group, with all the *V. anguillarum* serotype O1 genomes except 090819-1/28A. The remaining 8 clusters contained all of the *V. anguillarum* serotype O2A, O2B, O3 and a single O1 strain.

Neither the serotype O3 strain 4070 E nor the serotype O2A strain 850617-1/1 isolated from a Northern pike clustered with any other genomes. Three strains, one serotype O2B from an eel and two serotype O2A, made up the third group. The fourth cluster contained three O2A strains, of which two were isolated from European flounders. The serotype O2A genome 100719-1/3A did not group with other genomes in cluster five, but was most closely related to the strains in group six. Group six contained six serotype O2A genomes, all isolated from rainbow trout between 2004 and 2015. Two strains clustered distantly in group seven. One was a serotype O2A strain, while the other, surprisingly, was the serotype O1 genome 090819-1/28A, which was the only serotype O1 strain that did not cluster with the other serotype O1 genomes. The eighth group was the largest, with nine serotype O2A strains, isolated from rainbow trout and a cod in 1976–2009.

A total of 18.711.721 SNPs was found after all 44 genomes were mapped to the serotype O1 reference genome 775. A minimum of 41 and a maximum of 28.602 SNP differences between the individual genomes were detected, indicating *V. anguillarum* to be highly genetically diverse.

When divided into serotypes, the serotype O1 genomes had 526.525 SNPs with a minimum of 141 and a maximum of 27.036 within the group. The serotype O2A genomes had a total of 4.903.391 SNPs, with a range between 41 and 27.635 SNPs within the group. The only serotype O2B genome, 030305-1/5, had between 707 and 27.392 SNPs. The serotype O3 strain 4070 E was highly distinct from the other genomes under analysis, with 24.227 to 28.602 SNPs. The minimum number of 41 SNPs was found between strain 090707-1/2A and the two strains 090602-1/6B and 090707-1/1A, all serotype O2A strains. These three strains were all isolated in 2009, one in June and two in July, from the same facility, which explains the close phylogenetic relationship.

The maximum difference of 28.602 SNPs was found between the serotype O3 strain 4070 E isolated in 1978 and serotype O2A strain 100819-1/4A isolated in 2010, both isolated from rainbow trout.

### Pan-genome

The pan-genome of the 44 *V. anguillarum* genomes contained a total of 9.537 genes, which is more than previously reported (Holm et al., 2018; Castillo et al., 2017; Busschaert et al., 2015). To further investigate the difference between the *V. anguillarum* serotype O1 and O2A strains, the pan-genome was also calculated for these two groups separately. The results can be seen in Table 1.

**Table 1:** Pan-genome results for the three groups.

Pan-genome	44 Genomes	Serotype O1	Serotype O2A
Core genes (99% <= strains <= 100%)	2,694	3,408	2,788
Soft core genes (95% <= strains < 99%)	179	0	121
Accessory genes (15% <= strains < 95%)	2,455	1,442	2,645
Cloud genes (0% <= strains < 15%)	4,209	1,962	3,020
Total genes (0% <= strains <= 100%)	9,537	6,812	8,574

**Note:**

Pan-genome results for the three groups, as determined by Roary. The three columns represent the calculated pan-genome results for 44 genomes (all), the serotype O1 genomes (19) and O2A (23) genomes, respectively.

**Figure 2:**
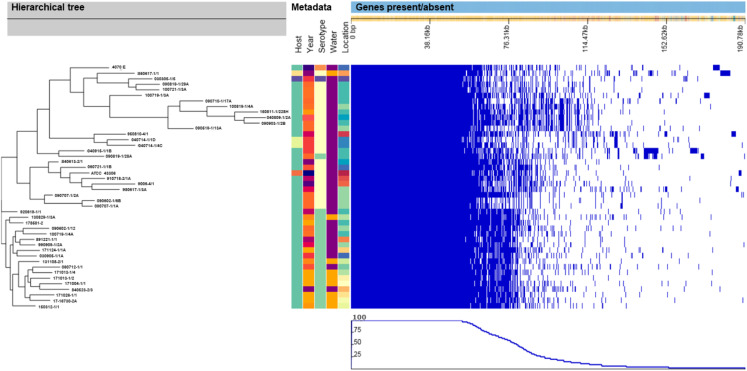
Hierarchical tree of genomes, metadata and a matrix with the presence and absence of the accessory genes. Hierarchical tree of genomes, as determined by Roary, compared to metadata and a matrix with the presence (blue blocks) and absence (white areas) of the accessory genes found in the pan-genome. Metadata details for each strain can be seen in Table S1.

Similar to the SNP-based phylogenic tree, the hierarchical tree based on the gene presence/absence in the accessory genome (Fig. 2) contained eight clusters that included all of the *V. anguillarum* serotypes O2A, O2B, O3 and the O1 genome 090819-1/28A. The distance between the genomes in these clusters was very variable, and they grouped in clades identical to those of the SNP-based tree. The rest of the serotype O1 genomes grouped into two clades, one consisting of only one strain, 920619-1/1, and the remaining serotype O1 strains, as a well uniform group in the other.

### Acquired antibiotic resistance genes

All of the strains contained acquired antibiotic resistance genes. Their distribution is shown in Fig. 1. Thirty-six strains contained the *qnrVC6* gene for resistance to fluoroquinolone/quinolone and the *tet(34)* gene for tetracycline resistance. The remaining eight strains only contained the *tet(34)* gene (Table S2) (910718-2/1A, 850617-1/1, 100721-1/3A, 090710-1/17A, 090707-1/1A, 090602-1/6B, 9006-4/1, and 4070 E). All of these strains were isolated from rainbow trout in the years between 1978 and 2010. Seven strains were serotype O2A, and the last one was the only O3 strain included in the investigation. The sequence similarity of *qnrVC6* genes was above 98%, and among *tet(34)* genes, above 82% in all positive strains.

### Plasmids

All of the 19 serotype O1 strains except one, 090819-1/28A, contained a pJM1-like plasmid (Table S3). Of these, three strains harbored the plasmid pjM1 and 15 had P65. Nine out of 23 serotype O2A strains contained the plasmid P15. The serotype O2B and O3 strains did not contain any of the nine plasmids investigated. The distribution of plasmids among the strains can be seen in Fig. 1.

### Virulence genes

More than 85% of the virulence-related genes included in the analysis were detected in the examined strains (Table S4). All of the genes were present in at least one strain, but no strain contained all of the virulence genes (between 213 and 234). In addition to the 250 virulence-related genes of *V. anguillarum* strains 775 and RV22 that we screened for, 17 other virulence-related genes were identified in the pan-genome data. Genes involved in iron transport, motility, chemotaxis, RTX toxins, type IV pilus, and quorum sensing were found in all of the strains. Although the distribution of virulence genes was rather similar across all the strains (Table S4), there was some evident variance between serotypes. The distribution of the 58 virulence genes that were not conserved can be seen in Fig. 1. No virulence genes were identified using the VirulenceFinder database.

All serotype O1 strains contained the autotransporter adhesion (*TAA*) gene (Rock & Nelson, 2006) and Mannose-sensitive haemagglutinin biogenesis protein *MshQ* gene (Floyd et al., 2020); none of these genes were found in the serotype O2 or O3 strains. Also, all O1 strains contained the lipase family protein gene (Rock & Nelson, 2006), the heme receptor *huvA* gene (Li & Ma, 2017), and the *toxA* gene (Reyes-Becerril et al., 2016); these genes were found in 8%, 71% and 52% of the O2 and O3 strains, respectively. None of the serotype O1 strains carried the heme receptor gene *huvS* (Mouriño et al., 2005), which were present in 20% of the O2 and O3 strains. Two RTX toxin genes also showed great variation between serotypes (Liu et al., 2009). Both were *apxIA* genes of approximately the same size, 4,533 and 4,479 bp, with an ID of 92.91% between them; *apxIA* was found in 21% of the serotype O1 strains, but in 88% of the serotype O2 and O3 strains, whereas *apxIA* group 1081 was found in 95% of the serotype O1 strains, but only in 12 % of the O2A and O3 strains. All strains except the serotype O2B 030305-1/5 lacked the *empA* gene (Rock & Nelson, 2006).

In general, serotype O1 strains were a uniform group, which only showed variation in a total of 12 virulence genes. The only exception was the O1 serotype strain 090819-1/28A, which did not have a plasmid, like the other serotype O1 strains in this study, and therefore lacked the iron-sequestering system consisting of 11 virulence genes. Instead 090819-1/28A carried ten genes encoding piscibactin biosynthesis, genes that was not found in any other serotype O1 strains, but in all serotype O2 and O3 strains, with the exception of 850617-1/1. The serotype O2 and O3 strains showed variation in 40 of the virulence genes.

### Antimicrobial sensitivity testing

Due to the high distribution of the *qnrVC6* and the *tet(34)* genes in the strains, all strains were tested for resistance to quinolone (oxolinic acid) and tetracycline (oxytetracycline). All strains were sensitive to both oxytetracycline and oxolinic acid (Table S2).

## Discussion

In this study, 44 *V. anguillarum* strains were sequenced, and the resulting genomes were subjected to a comparative genome analysis to assess the genetic diversity of particular serotypes O1 and O2A, originating from Danish rainbow trout.

In regards to genome properties, the genomes did show some variation as compared to the *V. anguillarum* genomes available in GenBank. For the genomes in this study, the genome size varied from 435,610 to 556,280 Mb, the total number of genes was 4,436–6,304, the total number of CDS was 4,364–6,225, and the number of proteins 4,225–6,025. This was in the higher spectrum compared to the *V. anguillarum* strains in GenBank with a genome size of 309,790–489,769 Mb, total numbers of genes 2,890–4,697, total numbers of CDS 2,793–4,560, and number of proteins 2,722–4,127. Likewise, for the strains in this study, the number of RNAs was 72–92 and the GC% was 43,74–44,42, which is in a lower spectrum than the genomes in Genbank, in which number of RNAs was 97–137 and the GC% was between 44,30 and 45,46. However, since the 44 genomes analyzed represent the largest collection of genome-sequenced *V. anguillarum* thus far and more than double the number of unique *V. anguillarum* genomes in GenBank, some variation is expected. In addition, the completeness of the genomes in GenBank differs a lot, which adds to the apparent variation. Also, the genomes in this study increase the number of O2 and O3 strains in GenBank by 62%, which is also likely to add some deviation.

Serotype O1 *V. anguillarum* strains are known to be a highly homogenous group, while serotype O2A strains are known to be a very diverse group (Pedersen, 1999; Austin et al., 1995; Hickey & Lee, 2017). Generally speaking, this concurs very well with our phylogenetic findings. Yet, one serotype O1 genome, 090819-1/28A, was atypical and did not group with the other serotype O1 strains in the SNP-based phylogenetic tree (Fig. 1). When this strain is excluded from the serotype O1 group, the total number of SNPs drops from 526.525 to 40.737 and the SNP span from 141–27.036 to 141–409 SNPs, clearly demonstrating the homogeneity within this group, which can also be seen in Fig. 1. In contrast, the group of serotype O2A strains is very diverse with a SNP span from 41 to 27.624, a total of 4.903.391 SNPs and serotype O2A strains was found in 7 of the 9 identified clusters. Since SNP analysis does not include information about sequences in the accessory genome, the pan-genome was calculated to add more information.

The pan-genome analysis revealed that the 44 *V. anguillarum* strains had 2,694 core genes. This is a slightly bigger core genome than the previously reported 2,370 genes, based on 28 genomes (Castillo et al., 2017) and 2,574 genes based on 11 complete genomes (Holm et al., 2018), especially because the number of genomes is considerably bigger in this study. However, the majority of the strains in this study were isolated from a few different hosts and from a very limited geographical area as compared to the *V. anguillarum* strains in the two mentioned studies and are therefore likely to be closer-related and share a larger proportion of genes. The accessory genome contained 2,455 genes, which is right in the middle of the earlier described 2,183 (Holm et al., 2018) and 2,870 (Castillo et al., 2017) genes for *V. anguillarum*. Only one other study reports the total number of unique genes in *V. anguillarum*, 2,910 (Holm et al., 2018), which is a lot less than the 4,209 unique genes that were found in this study. This might be explained by the fact that this study had four times more genomes in the analysis. As can be seen from these results, the total number of genes among the strains is much larger than those found for the individual strains, again suggesting considerable variation among strains.

When compared, the pan-genome of the serotype O1 strains contained 18% more core genes than the serotype O2A strains, whereas the serotype O2A strains had 46%, 35% and 21% more accessory, unique and total genes respectively than the serotype O1 strains. Again, the serotype O1 strains in this study are a very homogenous group, compared to the serotype O2A strains, which demonstrate much more diversity. The generated hierarchical tree and the matrix created based on the presence/absence of genes in the accessory genome (Fig. 2) also support this general difference between the serotypes. The great diversity within the *V. anguillarum* serotype O2A genomes is most likely the explanation why the commercially-available, and experimental vaccines based on only one serotype O2A strain, provide good protection from infection from some strains, but not for others (Marana et al., 2019; Mikkelsen et al., 2007). The above comparison with pan-genome analysis from other studies indicated that increased variation in host species and geographical location results in a smaller core and a bigger accessory genome. Hence, *V. anguillarum* strains from different host species and from diverse geographical locations are more prone to show variation than strains isolated from the same host species and a smaller geographical area. Also, in previous studies *V. anguillarum* genomes to some extent cluster with strains isolated from the same host species and/or isolated from the same country (Busschaert et al., 2015; Castillo et al., 2017). This emphasizes how unlikely it is to obtain good overall protection from a vaccine based on a random selected *V. anguillarum* strain, especially if it is not isolated from the targeted host species and geographical area. Unfortunately, it was not possible to gain access to the genome sequences, DNA, or the strains used in the commercial vaccine made for Norwegian salmon, so the variation between these and the Danish strains from rainbow trout could not be examined. However, based on the results in this study, it is evident, that a vaccine must be founded on strains, carefully selected to represent the genetic diversity within serotype O2A. A vaccine based on multiple carefully selected strains or perhaps a recombinant DNA vaccine, could be a solution to create better protection against *V. anguillarum* in Danish rainbow trout.

Besides for the obvious difference between the serotype O1 and O2/O3 strains, there does not appear to be any evident sign that the strains cluster together or not by year of isolation, location, host, or water type. However, there were a few trends worth mentioning. Not surprisingly, strains isolated from the same location within a narrow timeframe are more likely to be closer-related than strains from different facilities or with decades between isolation dates. In general, it seems like the serotype O1 strains from rainbow trout in fresh water cluster closer to each other than to strains isolated from fish in salt water; however, this trend is not consistent and might be biased by the fact that all the fresh water strains were isolated within a relatively narrow time period (2013–2017), multiple strains were taken from the same locations, and some locations received fish from the same provider. Also, the survival of this pathogen in freshwater environments is probably due to biofilm formation and not adaptation, as has been the case with vibriosis outbreaks in the ayu (*Plecoglossus altivelis*) in Lake Biwa, Japan (Eguchi, Fujiwara & Miyamoto, 2000; Frans et al., 2011).

The majority of serotype O1 *V. anguillarum* strains isolated from the Danish rainbow trout carry a pJM1-like plasmid (Pedersen, 1999) and 18 of the 19 serotype O1 strains in this study contained a pJM1-like plasmid. pJM1 is a 65-67 kilobasepair (kbp) plasmid found in serotype O1 *V. anguillarum* strains and is the most extensively studied plasmid within *V. anguillarum* (Pedersen, 1999; Hickey & Lee, 2017), first published by Crosa, Schiewe & Falkow (1977). The pJM1 plasmid has been described as an important part of the virulence of serotype O1 strains, due to the siderophore anguibactin encoded by this plasmid (Crosa, 1980; Pedersen et al., 1997). Yet, the presence of a pJM1-like plasmid is not essential for *V. anguillarum* serotype O1 strains to cause disease (Castillo et al., 2017). Castillo et al. (2017) suggest that production of the siderophore vanchrobactin might be responsible for virulence in serotype O1 strains without the pJM1 plasmid. But since all of the genes needed for vanchrobactin production were present in the 72 *V. anguillarum* strains analyzed in both this (Tabel S4) and Castillo’s study (Castillo et al., 2017), regardless of serotype, plasmid content and pathogenic potential, this is probably not the case. Recently, Balado et al. (2018) found evidence, that pathogenic *V. anguillarum* strains lacking the anguibactin system, produce two siderophores vanchrobactin and piscibactin. They also concluded that although both siderophores are produced simultaneously, piscibactin is the primary factor in virulence, while vanchrobactin plays a minor role (Balado et al., 2018; Thode et al., 2018). These results are very much in line with the findings in this study. All the serotype O2 and the O3 strains, with only one exception the O2A strain 850617-1/1, carried ten genes encoding piscibactin biosynthesis. This was also the case for the serotype O1 strain 090819-1/28A, while the rest of the serotype O1 strains, which contained a pJM1-like plasmid, did not. All the strains in our study were isolated from vibriosis outbreaks in Danish rainbow trout farms or from sick wild fish and are as such assumed to be pathogenic. Virulence experiments have, as far as the authors are aware, only been performed on strain 090819-1/28A, proving the capacity of the only strain without a pJM1-like plasmid to cause vibriosis in rainbow trout (Marana et al., 2019). Hence, supporting the hypothesis that piscibactin likely is the main virulence factor to infect fish for strains lacking the anguibactin system.

Nine of the 23 serotype O2A *V. anguillarum* strains isolated from cod and rainbow trout from 1980 to 2010 carried the plasmid p15. The p15, with 15 kbp, has recently been recognized for the first time, in the complete *V. anguillarum* serotype O1 genome VIB43, isolated from a sea bream (*Sparus aurata*) in Italy in 1991 (Holm et al., 2018; D. Austin, 2019, personal note). The plasmid contains 16 genes and 11 proteins, but the plasmid annotation report did not reveal any virulence-related genes. The only plasmid previously reported from a serotype O2 strain thus far is the considerably larger plasmid p292 (292 kbp), which was recently recognized in the first complete genome sequence of a *V. anguillarum* serotype O2 strain (Holm et al., 2018). This strain VIB12 was isolated from the sea bream in Greece in 1991 (D. Austin, 2019, personal note).

The findings of virulence-related genes in regards to type and numbers in each of the 44 *V. anguillarum* strains in this study is very similar to the findings of virulence-related genes in 28 *V. anguillarum* strains analyzed by Castillo et al. (2017). The *V. anguillarum* strains in the mentioned study were also screened for >200 virulence-related genes, and a total of 163 genes were present in all 28 strains (Castillo et al., 2017). In our study, 209 virulence-related genes were present in all strains, indicating that these strains are more similar when it comes to virulence factors. An explanation for this may be that almost all of the strains were isolated from Danish rainbow trout in Nordic countries, whereas the strains from the other study represent much greater diversity in terms of geographical locations and hosts (Castillo et al., 2017).

A clear difference in the content of virulence factors in serotype O1 and O2A strains was indeed found in our analyses, but this comparison has not been made in previous studies. These differences are depicted in Fig. 1 where only virulence factors that are not conserved are shown.

All of the 44 *V. anguillarum* strains contained the acquired antibiotic resistance gene *tet(34)* for tetracycline resistance in the chromosome. *tet(34)* is commonly found in *Vibrio*, and causes the activation of Mg2+-dependent purine nucleotide synthesis, which protects the protein synthesis pathway (Nonaka & Suzuki, 2002).

Tetracycline was used in Danish aquaculture starting in 1965 and phased out when other antibiotics became available in the mid-1980s (Dalsgaard, 2014). Currently, special permission is required to use tetracycline, and it is not used at all in Danish marine fish farms and only used in very limited amounts in freshwater farms (use in 2013, 2014 and 2015; 2, 0 and 0,7 Kg/year, respectively) (Ministry of Environment & Food of Denmark, 2017). However, tetracycline is still used frequently in aquaculture in other parts of the world for both treatment and prevention (Sub-Committee on Aquacultura, 2017; Ahsan, 2018). Acquired tetracycline resistance is a well-known problem in aquaculture, and tetracycline resistance genes are known to persist for years after the selection pressure has disappeared (Tamminen et al., 2011). Nevertheless, acquired antibiotic resistance is not exclusively triggered by the use of antibiotics. Bacterial resistance to heavy metals (e.g., copper, chromium, cadmium, arsenic) and antibiotics simultaneously increased with the presence of heavy metals alone (Pruden et al., 2006; Knapp et al., 2011; Tan et al., 2018); hence, water pollution can also be a source of antibiotic resistance development in *V. anguillarum*. For example, the *tet(34)* gene increased by 27.70 times in bacteria exposed to arsenic for six hours, and the co-selected resistance was well-maintained for up to seven days without selective pressure (Zhang et al., 2020). As such, our findings are not surprising, but they do not correlate very well with the fact, that all 44 strains were phenotypic sensitive to oxytetracycline. However, in a previous study, 25 *V. anguillarum* strains obtained from eight different marine rainbow trout farms in Denmark were tested for antimicrobial susceptibility, 24 of the 25 strains were sensitive and only one of the 25 showed intermediate sensitivity to tetracycline (Pedersen et al., 2008), which is in line with our findings. Recently, the *tet(34)* gene was also detected in the *Vibrio rotiferianus* genome SSVR1601, but surprisingly and similar to the results in this study, antimicrobial sensitivity testing indicated that the strain was sensitive to tetracycline (Zhang et al., 2019). However, it is possible that the gene is not expressed in the phenotype, due to lack of selection pressure.

It is also remarkable that not only the rainbow trout strains, but also the strains from wild and freshwater fish, carry the *tet(34)* gene, and it would be interesting to investigate how widespread the presence of this gene is in *V. anguillarum* in general.

Thirty-six strains contained a *qnrVC6* integron-mediated fluoroquinolone/quinolone resistance gene in the chromosome. *Qnr* proteins are pentapeptide repeat proteins that mimic DNA and protect the cell from the activity of fluoroquinolone/quinolone antibiotics and bacteria-carrying *qnr* genes present with decreased susceptibility to fluoroquinolones/quinolones (Fonseca & Vicente, 2013). Low-level resistance to fluoroquinolone antibiotics conferred by a *qnr* gene is associated with decreased bactericidal activity of ciprofloxacin in vitro and in vivo, similar to that obtained with a *gyrA* mutation (Allou et al., 2009; Jakobsen et al., 2012).

Fluoroquinolone has never been used, and oxolinic acid is the only quinolone in Danish aquaculture used since 1986 (Dalsgaard, 2014). In a Danish study, 417 *V. anguillarum* strains were phenotypic-tested for oxolinic acid resistance from 1980 to 2010. In total, 27 strains (6.5 %) were resistant. In 1989, three of 82 (3.6%) strains were resistant, and in 2009 three of 37 (8,1%) strains were resistant, hence showing a small increase in the number of oxolinic acid resistance *V. anguillarum* strains over time (Dalsgaard, 2014). All strains in this study were susceptible to oxolinic acid. The strains not carrying the *qnrVC6* gene are isolated from 1978-2010. All 11 strains isolated after 2010 contains *qnrVC6*, however, it is noteworthy that isolates from wild fish, as well as three of six strains isolated before 1986, in which oxolinic acid was first used, also carry the gene. This means that the *qnrVC6* gene was already present in Danish *V. anguillarum* strains before the use of quinolones and also in wild fish, where the selection pressure should be considerably lower than in the population treated with quinolones. The decreased susceptibility to fluoroquinolones/quinolones is as such not developed due to the use of quinolones in Danish aquaculture, but perhaps due to exposure to heavy metals available from natural environmental sources or through pollution (Pruden et al., 2006; Knapp et al., 2011; Tan et al., 2018). However, the use of quinolones probably induces an increased number of strains carrying the *qnrVC6* gene.

Previous genome studies of multiple *V. anguillarum* strains have not investigated the presence of acquired antibiotic resistance genes in the genomes. It would be interesting to research whether the *tet(34)* and *qnrVC6* genes also are common in *V. anguillarum* strains isolated outside the Nordic countries and from other host species than those included in this study.

The atypical serotype O1 strain 090819-1/28A did not group with the other serotype O1 strains in any of the phylogenetic trees. Instead, it grouped with the serotype O2A strain 040915-1/1B, isolated from a Danish rainbow trout in 2004. Both strains were isolated from the same facility. The two genomes had 3.436 SNPs, whereas 090819-1/28A had 26.966–27.036 SNPs with serotype O1 strains and 23.298–27.320 with the rest of the serotype O2A genomes. This was also the only serotype O1 genome without a plasmid. However, except for the lack of 11 virulence genes found on the plasmid and instead containing ten genes encoding piscibactin biosynthesis, 090819-1/28A showed a virulence pattern typical of serotype O1 strains (Fig. 1). As in the rest of the serotype O1 genomes, 090819-1/28A also contained both the *tet(34)* and *qnrVC6* genes.

Due to the unsuspected phylogeny results, 090819-1/28A was examined again to confirm the identity and serotype. However, this is not a unique occurrence. In a previous study, two serotype O1 strains also grouped with serotype O2 and O3 strains in phylogenetic trees based on both SNPs and the core genome (Castillo et al., 2017). Interestingly, both of these strains were also isolated from Danish rainbow trout and did not contain a pJM1-like plasmid.

## Conclusion

The genome sequences of the 44 strains analyzed in the present study more than doubles the number of publicly available unique *V. anguillarum* genome sequences. Also, the number of available serotype O2A genomes will increase from three to 26, subsequently providing a strong basis for future genetic studies of *V. anguillarum*.

The presence of acquired antibiotic resistance genes in *V. anguillarum* genomes had not been investigated in previous studies, but ARGs were found in all the strains included in this study. All strains carried the *tet(34)* gene for tetracycline resistance, and 36 strains also contained the resistance gene *qnrVC6* for increased fluoroquinolone/quinolone resistance. But interestingly, all strains were phenotypic sensitive to both oxytetracycline and oxolinic acid. As expected, almost all (18 of the 19) of the serotype O1 strains in this study contained a pJM1-like plasmid. Nine serotype O2A strains carried the plasmid p15, which had previously only been identified in a single serotype O1 *V. anguillarum* genome. Although the distribution of virulence genes was rather similar for all the strains, there was some evident variance between serotypes. Most significant, all the serotype O2 and O3 strains except one, as well as the only serotype O1 strain without a pJM1-like plasmid, carried genes encoding piscibactin biosynthesis. Hence supporting the hypothesis, that piscibactin plays a crucial role in virulence for pathogenic strains lacking the anguibactin system. The phylogenetic analysis and pan-genome calculations revealed great diversity within the species of *V. anguillarum*. Serotype O1 strains were in general very similar, whereas considerable variation was found among serotype O2A strains. The great diversity within the *V. anguillarum* serotype O2A genomes is most likely the reason why the commercially-available, as well as experimental, vaccines provide good protection from infection from some strains, but not from others. Hopefully, the new genomic information provided in this study might help develop an optimized vaccine against *V. anguillarum* in the future to reduce the use of antibiotics, minimize economic losses and improve the welfare of the fish.

## Supplemental Information

10.7717/peerj.10451/supp-1 Supplemental Information 1 Enlarged copy of the phylogenetic tree from Figure 1, including bootstrap values.

10.7717/peerj.10451/supp-2 Supplemental Information 2 Metadata and genome properties for *V. anguillarum* strains.

10.7717/peerj.10451/supp-3 Supplemental Information 3 Acquired antibiotic resistance genes in the 44 *V. anguillarum* strains.

10.7717/peerj.10451/supp-4 Supplemental Information 4 Plasmid content in the 44 *V. anguillarum* strains.

10.7717/peerj.10451/supp-5 Supplemental Information 5 Putative virulence factors content in the 44 *V. anguillarum* strains.

## Additional Information and Declarations

### DNA Deposition

The following information was supplied regarding the deposition of DNA sequences: Genbank accession numbers for the 44 genomes are available in Table S1.

### Data Availability

The following information was supplied regarding data availability: The raw data is available at figshare: Hansen, Mie Johanne (2020): Vibrio anguillarum raw genome data. figshare. Dataset. https://doi.org/10.6084/m9.figshare.12469061.v1
