# Supplementary figures and images for: Analysis of 44 Vibrio anguillarum genomes reveals high genetic diversity
Source: PeerJ. 2020 Dec 3;8:e10451. doi: 10.7717/peerj.10451 (PMC7719292; doi:10.7717/peerj.10451)

Enlargement of phylogenetic tree based on single nucleotide polymorphisms (SNPs) from Figure 1.

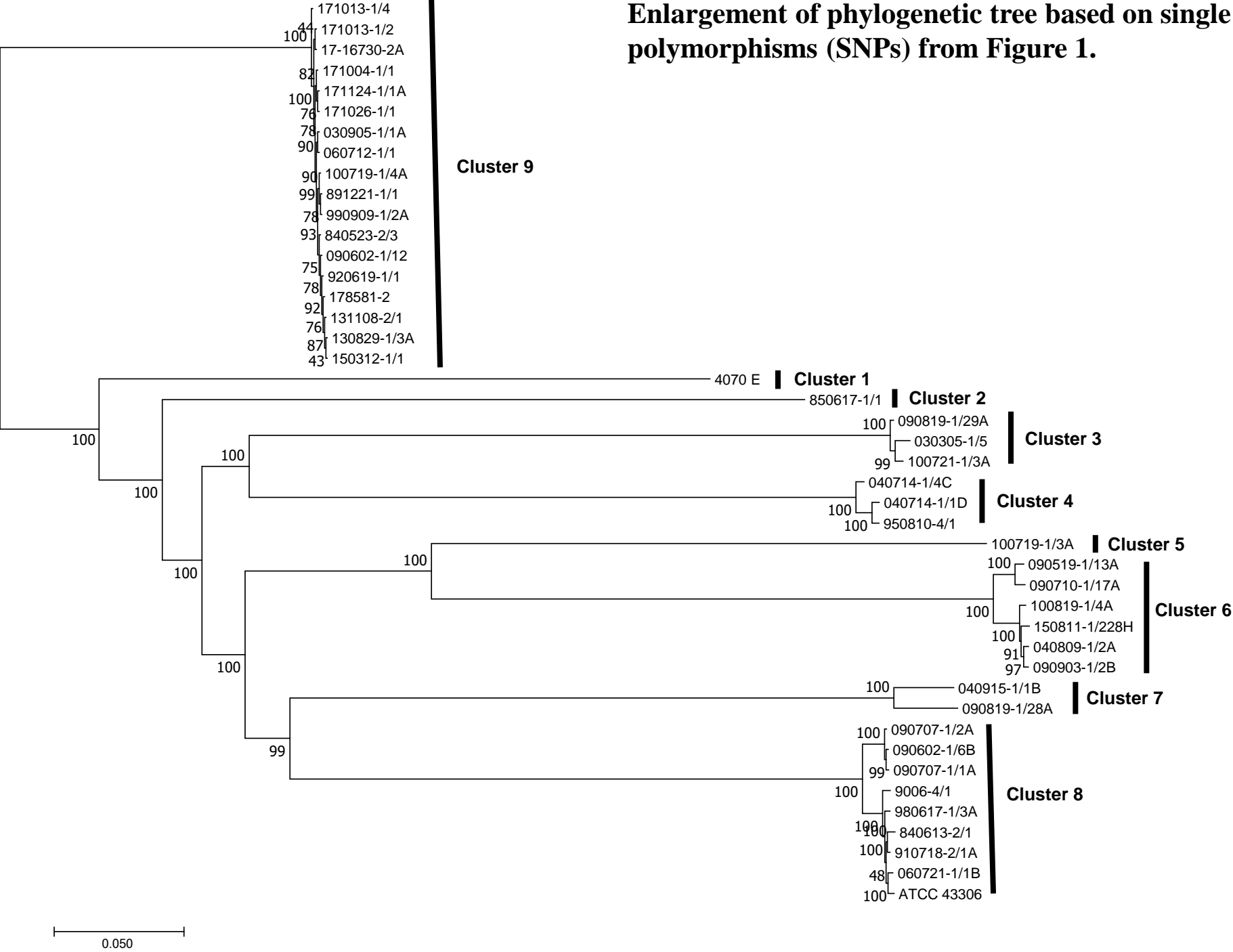

Supplement: Supplemental Information 1 [file peerj-08-10451-s001.pdf]
